# Boosting the Piezoelectric Sensitivity of Amino Acid Crystals by Mechanical Annealing for the Engineering of Fully Degradable Force Sensors

**DOI:** 10.1002/advs.202207269

**Published:** 2023-02-12

**Authors:** Yuanqi Cheng, Juan Xu, Lan Li, Pingqiang Cai, Ying Li, Qing Jiang, Wei Wang, Yi Cao, Bin Xue

**Affiliations:** ^1^ Collaborative Innovation Center of Advanced Microstructures National Laboratory of Solid State Microstructure Department of Physics Nanjing University Nanjing 210093 P. R. China; ^2^ Jinan Microecological Biomedicine Shandong Laboratory Jinan 250021 P. R. China; ^3^ Key Laboratory of Pharmaceutical Biotechnology Division of Sports Medicine and Adult Reconstructive Surgery Department of Orthopedic Surgery Drum Tower Hospital Affiliated to Medical School of Nanjing University Nanjing 210008 P. R. China; ^4^ Institute of Advanced Materials and Flexible Electronics (IAMFE) School of Chemistry and Materials Science Nanjing University of Information Science & Technology Nanjing 210044 P. R. China

**Keywords:** biodegradable, force sensors, peptides, piezoelectric, self‐assembly

## Abstract

Biodegradable piezoelectric force sensors can be used as implantable medical devices for monitoring physiological pressures of impaired organs or providing essential stimuli for drug delivery and tissue regeneration without the need of additional invasive removal surgery or battery power. However, traditional piezoelectric materials, such as inorganic ceramics and organic polymers, show unsatisfactory degradability, and cytotoxicity. Amino acid crystals are biocompatible and exhibit outstanding piezoelectric properties, but their small crystal size makes it difficult to align the crystals for practical applications. Here, a mechanical‐annealing strategy is reported for engineering all‐organic biodegradable piezoelectric force sensors using natural amino acid crystals as piezoelectric materials. It is shown that the piezoelectric constant of the mechanical‐annealed crystals can reach 12 times that of the single crystal powders. Moreover, mechanical annealing results in flat and smooth surfaces, thus improving the contact of the crystal films with the electrodes and leading to high output voltages of the devices. The packaged force sensors can be used to monitor dynamic motions, including muscle contraction and lung respiration, in vivo for 4 weeks and then gradually degrade without causing obvious inflammation or systemic toxicity. This work provides a way to engineer all‐organic and biodegradable force sensors for potential clinical applications.

## Graphical abstract

A mechanical‐annealing strategy is used to produce large scale amino acid films with well‐aligned crystalline phase and engineer all‐organic biodegradable piezoelectric force sensors. The mechanical‐annealed crystal film exhibits significantly boosted piezoelectricity and the packaged force sensors can be implanted in vivo for long‐term monitoring of dynamic physiological activities without additional invasive removal surgeries.

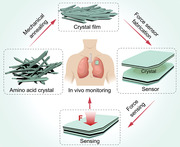

## 1 Introduction

Biomedical force sensors that can monitor biophysiological pressures and weak movements are promising for various biomedical applications, including early assessment and diagnosis of chronic and acute diseases in vivo as well as health‐care monitoring and long‐term recovery tracking.^[^ ^1^ ^]^ Especially for in vivo applications, the sensors are required to be implantable, flexible, biocompatible, and biodegradable to avoid inflammation, adverse immune response, and tissue damage due to the invasive removal surgery.^[^ ^2^ ^]^ Among various force sensors, piezoelectric sensors are advantageous because of their high sensitivity, ease of fabrication, and especially no need of battery power.^[^ ^3^ ^]^ Currently, most piezoelectric force sensors designed for biomedical engineering still comprise inorganic piezoelectric crystals, such as lead zirconate titanate^[^ ^4^ ^]^ or undegradable synthetic polymers, such as polyvinylidene fluoride,^[^ ^5^ ^]^ which extremely limits their real clinical applications. Recently, several degradable force sensors based on biodegradable and biocompatible piezoelectric poly l‐lactide (PLLA) have been developed,^[^ ^6^ ^]^ while inorganic elements such as metallic conductive layers were still used as electrodes,^[^ ^7^ ^]^ which certainly affects their complete biodegradation.^[^ ^8^ ^]^ It remains challenging to engineer all organic and fully degradable piezoelectric force sensors for in vivo biomedical applications.

Recently, many biomaterials with inherent biocompatibility and degradability have been discovered to show excellent piezoelectric responses.^[^ ^9^ ^]^ As one of the piezoelectric biomaterials, amino acid crystals^[^ ^10^ ^]^ are of special interest because they can be easily prepared with well‐defined crystalline phases and tunable self‐assembled structures.^[^ ^11^ ^]^ However, amino acid or peptide crystals growing under normal conditions exist as fragile small crystals of sizes in a range of micrometers to millimeters. Although some single crystals exhibit a piezoelectric coefficient as high as 10 pC N^−1^ along preferred directions,^[^ ^10a^ ^]^ macroscopic peptide powders often possess much reduced piezoelectric properties due to the random orientation and loose packing of the crystals. For the same reason, the crystals are difficult to fully contact with the electrodes, which impedes charge accumulation to the electrodes and leads to relatively low output voltages. Because peptide crystals are usually rigid and brittle,^[^ ^12^ ^]^ it remains a technical challenge to assemble large‐scale (larger than millimeters) films made of aligned amino acid crystals for piezoelectric force sensors.^[^ ^3d^ ^]^

Here, we present an all‐organic degradable piezoelectric force sensor based on mechanical‐annealed amino acid crystals. Using isoleucine as the model amino acid, we demonstrate stress‐induced alignment of small crystals to form large‐scale films with well‐aligned piezoelectric crystalline phases (defined as mechanical annealing).^[^ ^13^ ^]^ We further integrate the amino acid crystal films with conducting polyaniline (PAN) electrodes, and biodegradable poly(lactic acid) (PLA) coating layers to fabricate packaged force sensors. The resulting force sensors exhibit a broad sensing range (0.1–100 N), high sensitivity (<1 kPa), and reliable long‐term force sensing in physiological environments (>4 weeks). As a proof‐of‐principle demonstration, we implant the sensors into model animals and use them to monitor dynamic physiological activities, including muscle contraction and lung respiration for 4 weeks. More importantly, we demonstrate that the force sensors are biocompatible and fully biodegradable. Our work represents a key step toward engineering all‐organic and fully degradable force sensors for practical biomedical applications using amino acid crystals as piezoelectric materials.

## 2 Results and Discussion

### 2.1 Single Amino Acid Crystal and Mechanical Annealing of Amino Acid Crystals

**Figure 1:**
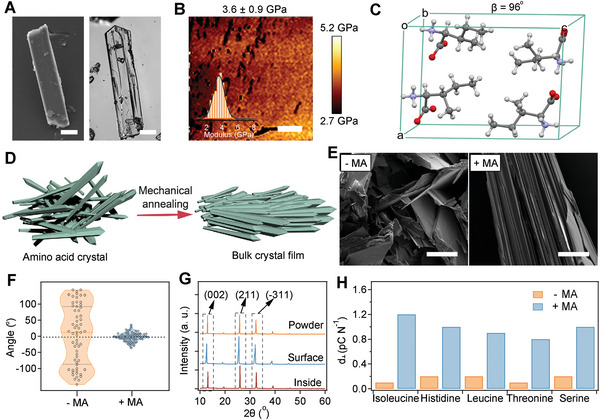
Characterization of isoleucine single crystals and mechanical‐annealed isoleucine crystals. A) SEM image (left) and microphotograph (right) of the isoleucine single crystal. Scale bar = 20 µm. B) Typical topographic modulus map of isoleucine single crystals based on AFM nanoindentation. The inset corresponds to the statistical Young's modulus distribution. Scale bar = 1 µm. C) Atomic packing and the monoclinic angle (*β*) of isoleucine single crystals. D) Schematic of the mechanical annealing of isoleucine crystals. The alignment of the crystals is significantly enhanced after mechanical annealing. E) SEM images of the isoleucine crystal without (− MA) and with (+ MA) mechanical annealing. F) Distribution of angles between the crystal's long axis and the horizontal *XY* plane (the plane perpendicular to the applied force) without (− MA) and with (+ MA) mechanical annealing. G) XRD of powder‐formed isoleucine single crystals and mechanical‐annealed isoleucine crystals (surface and inside). H) Quasistatic piezoelectric (*d*_⊥_) of different amino crystals (isoleucine, histidine, leucine, threonine, and serine) without (− MA) and with (+ MA) mechanical annealing.

We first prepared single isoleucine crystals as the starting materials. The isoleucine amino acid crystals were grown by gradually evaporating ≈80% of the water content of a condensed isoleucine solution (30 mg mL^−1^) at 60 °C. Scanning electron microscopy (SEM) and optical microscopy (**Figure** **1**A) revealed that isoleucine amino acid formed elongated small crystals. The length and thickness of the crystals were ≈10–100 and 4–9 µm, respectively. The Young's modulus and stiffness of the crystals measured using atomic force microscopy (AFM)‐based nanoindentation were 3.6 GPa and 68.2 N m^−1^ (Figure 1B and Figure S1, Supporting Information), respectively, comparable to those of highly oriented pyrolytic graphite,^[^ ^14^ ^]^ indicating that the crystals were stiff and brittle. Thermogravimetric and differential scanning calorimetry studies indicated that the isoleucine crystals were thermally stable, with a decomposition temperature of 283 °C (Figure S2, Supporting Information). The atomic packing of the isoleucine single crystal is shown in Figure 1C and the monoclinic angle (*β*) is ≈96°, consistent with the previous report.^[^ ^10a^ ^]^ Isoleucine crystalized with two independent nonplanar isoleucine molecules in the asymmetric unit (Figure S3, Supporting Information). Each isoleucine molecule formed seven hydrogen bonds with neighboring molecules, suggesting that hydrogel bonding is the main driving force for isoleucine packing. The asymmetric molecular packing and weak intermolecular interactions indicate that the crystals can couple mechanical stress to electrical polarization, thus showing piezoelectric properties.

Then, we aligned the amino acid crystals to form compact films by a mechanical‐annealing approach. We anticipated that the preferred orientation of crystals would be generated under mechanical stress, thus forming ordered structures in bulk films (Figure 1D).^[^ ^15^ ^]^ Upon compression, the crystals with the long axis not on the horizontal plane experienced higher stress than those on‐plane ones and were fractured. Thus, when the fractured crystals regrew, they tended to grow along the horizontal plane to avoid stress‐concentration. High pressure also promoted the formation of large crystals, as they are more stable than small ones under the same stress.^[^ ^16^ ^]^ Residual water on the crystal surface can facilitate the mechanical‐annealing process by accelerating the dissolution–recrystallization kinetics. We applied a pressure of 250 bar to the amino acid crystal powders (with ≈13% residual water) to prepare the piezoelectric film (Figure S4, Supporting Information). SEM images indicated that the mechanical‐annealed crystals (+ MA) exhibited lamellar structures at the mesoscale (right of Figure 1E and Figure S5A, Supporting Information), similar to the lamellar structure of shells.^[^ ^17^ ^]^ The surface of mechanical‐annealed crystals was flat (left of Figure S5B, Supporting Information), which enables tight contact with electrodes and coating layers to facilitate next‐step device fabrication. Moreover, the mosaic‐like patterns can be observed on the surface of crystal films, indicating the fracture and regrowth of single crystals during the preparation (right of Figure S5B, Supporting Information). In sharp contrast, the crystal powders without mechanical annealing (− MA) showed disordered structures (left of Figure 1E and Figure S5C, Supporting Information). The orientation of the crystals was concentrated to the horizontal *XY* plane (the plane perpendicular to the applied force), as the angles between the crystal's long axis and the *XY* plane were close to zero (Figure 1F), which is in sharp contrast to the random orientation of the peptide crystal powders without mechanical annealing. X‐ray diffraction (XRD) of the crystals revealed that the intensity ratios of the (211) and (002) peaks of the crystal films (both the surface and inside) increased by ≈50% compared with those of the crystal powders, suggesting that the (211) facet was the preferred orientation of the crystals after mechanical annealing (Figure 1G). The mechanical‐annealing strategy can also be extended to other amino acid crystals (i.e., histidine, leucine, threonine, and serine). Both the SEM images (Figure S6, Supporting Information) and the XRD patterns (Figure S7, Supporting Information) confirmed the formation of aligned lamellar crystalline phases after mechanical annealing. We then characterized the piezoelectric properties of the mechanical‐annealed crystal films by a d_33_ meter (350 µm in thickness, Figure 1H and Figure S8, Supporting Information). The measured piezoelectric constant in the normal direction of the film was defined as *d*_⊥_ hereafter and piezoelectric constants (*d*_⊥_) of all the amino acid crystals increased sharply upon mechanical annealing. Especially, the piezoelectric constant (*d*_⊥_) of the isoleucine crystal films increased from 0.1 to 1.2 pC N^−1^. We attributed the enhanced piezoelectric properties to the ordered alignment of the crystalline phase after mechanical annealing. Thus, we established a general method to engineer amino acid crystal films with excellent piezoelectric properties suitable for next‐step device fabrication.

### 2.2 Fabrication, Characterization, and Force Sensing Performance of the Packaged Force Sensor

**Figure 2:**
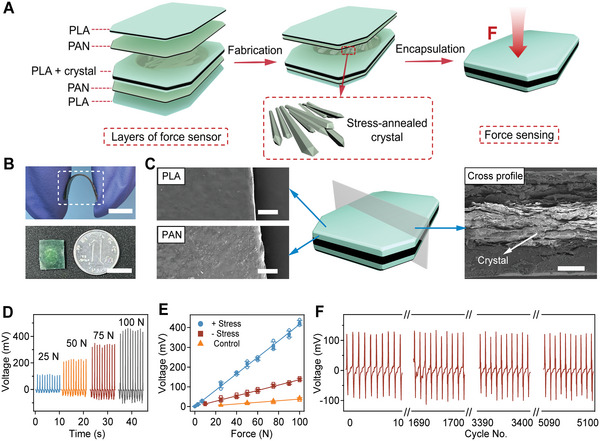
Fabrication, characterization, and force sensing of packaged force sensors. A) Illustration of the packaged force sensor and various components. Polylactic acid (PLA), polyaniline (PAN), and the mechanical‐annealed isoleucine crystal film are used to fabricate the encapsulation layer, conductive layer, and piezoelectric layer, respectively. B) Optical images of the force sensor held by fingers (top) and comparison with a dime coin (bottom). The scale bars are 5 (top) and 10 mm (bottom), respectively. C) SEM images of different layers (PLA, PAN, and crystal) of the force sensor. The crystal layers were sandwiched between PLA–PAN layers. Scale bar = 200 µm. D) Open‐circuit voltage of the force sensors containing the mechanical‐annealed isoleucine crystal film under different applied forces (25, 50, 75, and 100 N). E) Linear fitting of the open‐circuit voltage of the force sensors as a function of the applied force. The sensors were prepared using the mechanical‐annealed isoleucine crystal film (+ MA), unaligned isoleucine crystal powders (− MA), and without crystals (control). Values represent the mean and standard deviation (*n* = 3 independent samples). F) Long‐term sensing of the force sensor prepared using the mechanical‐annealed isoleucine crystal film for more than 5000 cycles under 25 N impulse impact.

After confirming the successful preparation of amino acid crystal films with high piezoelectric properties, we next fabricated all‐organic biodegradable packaged force sensors. **Figure** **2**A illustrates the sensor structure, which includes a layer of mechanical‐annealed isoleucine crystal film as the piezoelectric sensing unit, two layers of conductive PAN electrodes, and is fully covered with PLA to avoid leaking in the aqueous environment. The force sensors were only ≈0.7 mm thick and could be bent to more than 150° without fracture (top of Figure 2B). The width and length were both 10 mm (bottom of Figure 2B). SEM images revealed that the surfaces of the PAN and PLA layers were uniform and flat without noticeable defects and different layers were compactly integrated with each other (Figure 2C). The middle amino acid crystal layer formed tight and ordered lamellar structures, similar to unpacked films. Moreover, the lamellar structures of the crystal stacking allowed crystal sheets to slide along the horizontal plane and recover after the release of deformation, endowing crystal films with the ability to bend and recover under the assistance of PAN‐PLA covering. The fracture strength, tear strength, and fracture strain of the PAN‐PLA layers were 40, 1.2 MPa, and 37%, respectively, providing the sensors with high mechanical stability and flexibility (Figure S9, Supporting Information).

Next, we explored the force sensing performance of the packaged sensors containing mechanical‐annealed isoleucine crystal films. The open‐circuit voltage output was monitored upon applying impulse forces at 1 Hz. The voltage outputs were at the same frequency as the input signals, and the voltage outputs increased with increasing forces (Figure 2D). The force sensors showed fast responses and can sense in a broad frequency range from 0.5 to 10 Hz (Figure S10, Supporting Information). The output voltages for the sensors with mechanical‐annealed crystal films were about approximately threefold higher than those of the force sensors with unaligned isoleucine crystal powders (Figure 2E and Figures S11 and S12, Supporting Information). Moreover, voltage outputs of the sensors can be clearly detected even under an applied force as low as 0.1 N (1 kPa), indicating the high sensitivity of the force sensors (Figure S11A, Supporting Information). The voltage outputs exhibited an excellent linearity coefficient of determination (*R* ^2^ > 99%) over a broad force range (Figure 2E). Note that without the amino acid crystals, the voltage outputs of the electrodes and the laminate layers alone were negligible, suggesting that the piezoelectric response was mainly attributed to the mechanical‐annealed isoleucine crystal films (Figure 2E and Figure S13, Supporting Information). In addition, the force sensors showed reliable long‐term sensing for more than 5000 cycles under a 25 N impulse impact (Figure 2F). In contrast, the force sensors made of amino acid crystal powders were prone to generate permanent deformation under continuous impulse, thus showing reduced long‐term force sensing stability (Figure S14, Supporting Information). Notably, similar trends of the enhanced open‐circuit voltage outputs were observed in the force sensors fabricated using the mechanical‐annealed histidine crystals (Figure S15, Supporting Information), indicating the consistent enhancement of piezoelectric responses from mechanical annealing of different amino acid crystals.

### 2.3 In Vivo Sensing of Dynamic Physiological Signals

**Figure 3:**
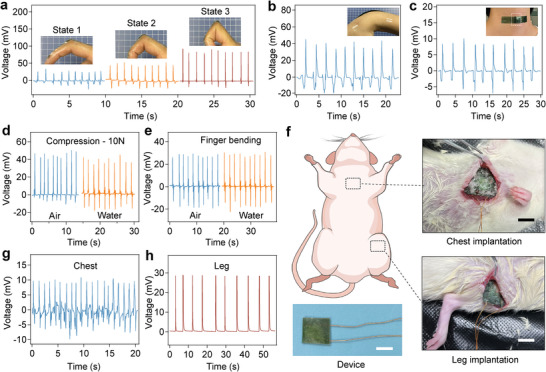
In vitro and in vivo performance of the packaged force sensors. A) Open‐circuit voltage of the force sensor fixed on a human finger upon bending in three states (frequency: 1 Hz). Insets show the movements of the finger bearing the sensor. B) Open‐circuit voltage of the force sensor fixed on a human wrist upon bending. The inset shows the fixing of the force sensor on the wrist. C) Open‐circuit voltage of the force sensor fixed on a human neck upon swallowing. The inset shows the fixing of the force sensor on the neck. D) Open‐circuit voltage of the force sensor in air and water obtained under 10 N impulse impact. E) Open‐circuit voltage of the force sensor fixed on a human finger upon bending in air and water. F) Illustration and photographs of the implantations of the force sensors in the chest and leg areas of Sprague–Dawley rats. Scale bars = 5 mm. G) Open‐circuit voltage of the force sensor implanted on the chest driven by respiration. H) Open‐circuit voltage of the force sensor implanted on the leg driven by gentle stretching.

After verifying the accuracy and reliability of the packaged force sensors, we next used them for monitoring dynamic motions in vitro. The force sensors were attached to the forefingers by flexible tape to monitor finger motions (**Figure** **3**A). Obviously, the sensor could precisely respond to different levels of bending and provide reliable measurements for multiple cycles. The output voltage increased from 25 to 100 mV with the increasing bending angle of the finger. Meanwhile, the flexibility and high sensitivity of the force sensor make it possible to sense the small angle change of wrist bending as well as the gentle motion of swallowing (Figure 3B,C). Furthermore, the PLA encapsulation of the sensor allowed the device to function well in aqueous environments (Figure 3D,E). The open‐circuit voltage of the force sensors underwater remained almost the same as that in air, indicating the excellent leak‐proof performance in aqueous environments.

Next, we evaluated the in vivo sensing performance of the implanted sensors in adult Sprague–Dawley rats. The force sensors (dimension: 8 × 8 mm, thickness: 0.7 mm) were implanted under the skin in the chest and leg area (Figure 3F). Two insulated wires were connected to the PAN layers of the device to monitor the output signals and avoid the interference of bioelectrical signals (Figure S16, Supporting Information). Sharp and separated voltage signals of ≈10 mV at frequencies of 60–70 min^−1^ were observed in response to rat respiration (Figure 3G and Movie S1, Supporting Information), which was comparable to or even superior to those of other organic flexible nanogenerators reported in previous literature.^[^ ^2^, ^18^ ^]^ Moreover, a voltage output of ≈30 mV was generated by the implanted force sensor when the rat leg was gently stretched and released cyclically (Figure 3H and Movie S2, Supporting Information). These results clearly demonstrated the sensing of dynamic physiological signals in vivo and the potential clinical applications of the packaged force sensors.

### 2.4 Biocompatibility

**Figure 4:**
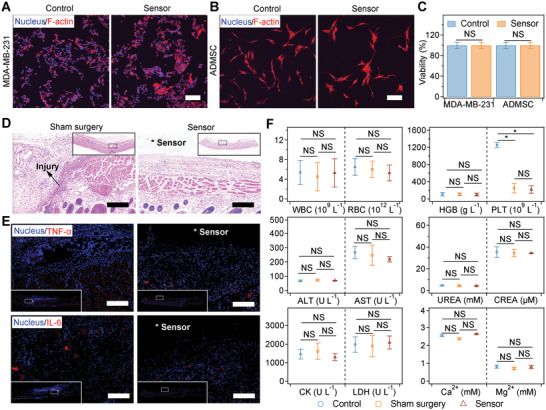
Biocompatibility of the force sensor in vitro and in vivo. A,B) Cellular morphology of MDA‐MB‐231 (A) and ADMSC (B) cells cultured in the presence of force sensors. The cytoskeleton and nucleus of cells were stained with phalloidin (red) and DAPI (blue) after 24 h of culture. Scale bar = 100 µm. C) Cell viability of MDA‐MB‐231 cells and ADMSCs cultured in the presence of force sensors after 24 h. Statistical significance was determined by a two‐tailed *t*‐test. NS, not significant. D) Representative histological images stained with hematoxylin and eosin (H&E) in vivo after subcutaneous implantation for 1 day. * represents the location of the force sensor. Scale bar = 200 µm. E) Representative immunostaining images of the sham surgery (left) and sensor‐implanted groups (right) identified by molecular markers (TNF‐*α* and IL‐6, colored in red). Cell nuclei were indicated by DAPI (blue). * represents the location of the force sensor. Scale bar = 200 µm. F) Hematological examination and blood biochemistry of the rats after subcutaneous implantation for 1 day. The blood of rats without any surgery was used as the control. WBC: white blood cell count, RBC: red blood cell count, HGB: hemoglobin, PLT: platelets, ALT: alanine transferase, AST: aspartate transferase, UREA: urea, CREA: creatinine, CK: creatine kinase, LDH: lactate dehydrogenase. Reported values represent the mean and standard deviation (*n* = 3 independent animals). Statistical significance was determined by a two‐tailed *t*‐test. NS, not significant. *p < 0.05.

Next, we investigated the biocompatibility of the packaged force sensors both in vitro and in vivo. For the in vitro cytotoxicity tests, human breast cancer cells (MDA‐MB‐231) and human adipose derived stem cells (ADMSCs) were chosen to act as the cancer and normal cell models to explore the response of different types of cells on the packaged force sensors. As shown in **Figure** **4**A,B and Figure S17, Supporting Information, the cytoskeleton and nucleus of MDA‐MB‐231 cells and ADMSCs cultured in the presence of the force sensors for 24 and 48 h were similar to those of the control groups, revealing that cell morphology, distribution, and density were not affected. Nearly no dead cells could be observed in the presence of force sensors for both cells, and the viabilities were higher than 99% (Figure 4C and Figure S18, Supporting Information). Moreover, similar results were observed for cells cultured in the presence of mechanical‐annealed isoleucine crystals, indicating the biocompatibility of isoleucine (Figure S19, Supporting Information).

The in vivo biocompatibility of the sensors was evaluated based on dorsal subcutaneous implantation in a rat model (Figure 4D–F). The sham surgery group was used for comparison. As indicated by the histological assessment (hematoxylin and erosion [H&E] staining), the force sensor did not cause any major damage to the surrounding dermal and muscular layers after subcutaneous implantation for 1 day (Figure 4D). Both groups showed mild inflammation without a significant presence of lymphocytes or an eosinophilic response. A low expression level of tumor necrosis factor‐*α* (TNF‐*α*) and interleukin‐6 (IL‐6) similar to that of the sham surgery around the sensor was observed according to the immunofluorescence staining (Figure 4E and Figure S20, Supporting Information), further indicating a mild inflammatory response. The acute systemic toxicity of the sensors was studied by hematological examination (white blood cell count, red blood cell count, hemoglobin, and platelet [PLT] count) and blood biochemistry (alanine transferase, aspartate transferase, urea, creatinine, creatine kinase, lactate dehydrogenase, Ca^2+^, and Mg^2+^). The similar levels of these indicators as those of the sham operation and control groups demonstrated that the implantation of sensors did not lead to systemic inflammation or affect the function of major organs (Figure 4F). The decrease in PLTs was probably due to the postsurgery coagulation. Similarly, no obvious inflammatory response or acute systemic toxicity was observed for the dorsal subcutaneous implantation of the mechanical‐annealed isoleucine crystals (Figures S21 and S22, Supporting Information). Moreover, the long‐term inflammation and systemic toxicity of the device after implantation for 6 weeks was negligible according to the H&E staining, hematological examination, and blood biochemistry (Figures S23 and S24, Supporting Information). Meanwhile, no obvious histologic response or pathological change to the major organs (heart, liver, spleen, lung, kidney, and stomach) was observed (Figure S25, Supporting Information). All these in vitro and in vivo results confirmed the biocompatibility of the force sensors.

### 2.5 Long‐Term Stability and Biodegradability

**Figure 5:**
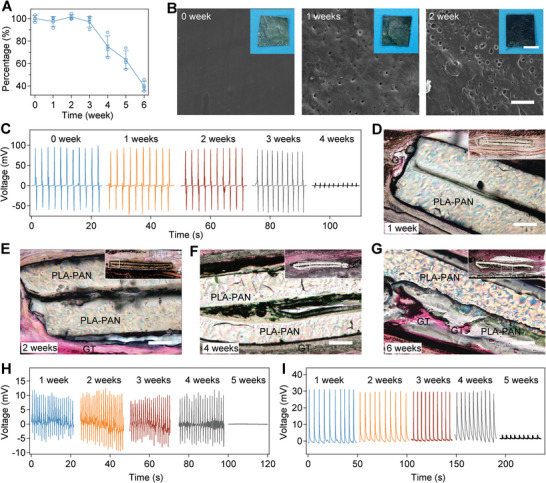
Biodegradability and long‐term sensing stability of the force sensors in vitro and in vivo. A) Remaining mass percentage of the force sensors incubated in simulated body fluid (SBF). Values represent the mean and standard deviation (*n* = 3 independent experiments). B) SEM images of the in vitro biodegradation of the force sensors in SBF. Insets are the corresponding photographs of the force sensors. Scale bars of the SEM and insets are 50 µm and 5 mm, respectively. C) Open‐circuit voltage of the force sensor driven by gentle compressions (20 N) after incubation in SBF for different times (0, 1, 2, 3, and 4 weeks). D–G) Representative histological images stained with H&E for assessment of the in vivo biodegradation of force sensors after subcutaneous implantation for 1 (D), 2 (E), 4 (F), and 6 (G) weeks. The sensors and surrounding tissues were resin embedded to avoid the sensor falling out during the staining. GT indicates granulation tissues. Scale bars = 200 µm. H,I) Open‐circuit voltage of the force sensors driven by respiration (H) and gentle stretching (I) in vivo after being implanted for different times (1, 2, 3, 4, and 5 weeks).

The packaged force sensors are expected to show excellent biodegradability due to the fabrication with all organic and degradable components. The in vitro degradation of force sensors was investigated by incubating the devices in simulated body fluid (SBF) at 37 °C for 6 weeks. The weight of the sensor decreased sharply from the 4th week (**Figure** **5**A), probably due to that the damages of PLA–PAN layers led to the leakage of amino acid crystals. The sensors lost more than 60% of their original weights in 6 weeks. Obvious porous structures appeared since the 2nd week and the sensor completely broke after 6 weeks (Figure 5B and Figure S26, Supporting Information). As indicated by the sensing evaluation after incubation for different times, the amplitude of the open‐circuit voltages remained almost unchanged in the first 3 weeks and significantly reduced since the 4th week (Figure 5C). Considering that the sensing ability of the sensors was mainly attributed to the mechanical‐annealed crystal, the sharp decrease from the 4th week was consistent with the in vitro degradation in SBF.

We also evaluated the in vivo biodegradability and long‐term sensing stability of the force sensors in a rat model (Figure 5D–I). After being implanted subcutaneously in the rat model for different times, the sensors and surrounding tissues were studied with histological assessment. Resin embedding was used to avoid the sensor falling out. The crystals inside the polymer layers completely dissolved into organic solvents during the preparation of the H&E staining. As indicated by Figure 5D–G, the force sensor degraded gradually in vivo. The cracks of the PLA–PAN layers increased with incubating time in vivo. The PLA–PAN layers were obviously thinner and penetrated through by granulation tissues after 6 weeks, indicating the breakdown of the sensors and the healing of the damaged tissues. The degradation rate in vivo was lower than that in vitro, probably due to the limited volume of the tissue fluid. Moreover, both the sensing of rat respiration and leg stretching remained consistent after implantation for 4 weeks and then declined at the 5th week, consistent with the trend of biodegradation in vivo (Figure 5H,I). These results revealed the excellent biodegradability and long‐term sensing stability of packaged force sensors in vitro and in vivo, as expected.

### 2.6 Discussion

Amino acid crystals are attractive piezoelectric materials due to their unique advantages, including ease of preparation, tunable self‐assembling structures, excellent biocompatibility, and controllable biodegradability. However, the as‐prepared amino acid crystals exist as randomly oriented and loosely packed fragile small crystals, greatly hindering the coupling of mechanical stress to electrical polarization and interfacing with electrodes. It remains challenging to produce bulk films with well‐aligned crystalline phases and smooth surfaces for practical device applications. Recently, Wang and coworkers elegantly demonstrated the use of polyvinyl alcohol (PVA)‐assisted self‐assembly to synthesize wafer‐scale piezoelectric films with aligned glycine crystal phases.^[^ ^3d^ ^]^ However, the existence of the PVA layers may reduce the open‐circuit voltages due to effects on electric contact with the electrodes and the high resistance.^[^ ^19^ ^]^ Although the film showed a high piezoelectric response, the resulting force sensors demonstrated low piezoelectric output voltages in vivo even when connected by highly conductive metallic electrodes. Here, we introduced a mechanical‐annealing strategy to produce well‐ordered isoleucine crystal materials at large scales for the first time. This method is general and can be extended to engineer other biomaterial‐based piezoelectric films. The alignment of the crystal phase in the films significantly boosted the piezoelectric constant at the bulk level and the flat surfaces enabled compact connection with conductive polymer electrodes. Therefore, the packaged force sensors showed high sensitivity and a wide force sensing range. Recently, many new types of biomolecular crystals with higher piezoelectric constants than the one used in this study have been explored using peptides and their derivatives.^[^ ^11^, ^20^ ^]^ Considering the universality of mechanical annealing, it is possible to further improve the performance of amino acid‐based biodegradable force sensors by replacing isoleucine with new piezoelectric biomaterials. Our study represents an important step toward engineering amino acid‐based piezoelectric devices for practical biomedical applications.

## 3 Conclusion

In summary, we presented a strategy for fabricating all‐organic degradable piezoelectric force sensors using mechanical‐annealed amino acid crystals. The crystals exhibited significantly boosted piezoelectric responses due to their ordered alignments at the mesoscopic scale after mechanical annealing. By coupling with conductive polymer electrodes and encapsulating them using PLA, the packaged force sensors showed a wide force sensing range, high sensitivity, and reliability for long‐term sensing in aqueous environments. In addition, all the materials used to fabricate the packaged force sensors were biocompatible and biodegradable. Thus, they could be used as implantable devices in vivo to monitor important physiological signals, such as muscle contraction and respiration, without invasive removal surgeries. Our work provides a general strategy to engineer all‐organic and biodegradable force sensors with amino acid crystals as piezoelectric materials for practical biomedical and clinical applications.

## 4 Experimental Section

### Preparation of Amino Acid Crystals and Mechanical‐Annealed Amino Acid Crystals

For the preparation of the isoleucine crystal (Figure S4A, Supporting Information), isoleucine was dissolved in deionized water to a concentration of 30 mg mL^−1^ and heated to 65 °C for 5 min. Then the solution was transferred to an ice water bath and allowed to stand for 6 h until the crystal nucleus was grown. Afterward, the solution was evaporated and crystallized in an oven at 60 °C until the volume of the liquid decreased by 80%. Finally, the crystal was collected and dried.

To achieve mechanical‐annealed crystal films (Figure S4B, Supporting Information), the as‐prepared crystal was filled into a tablet mold (circle tablet with a diameter of 8 mm and thickness of 1.5 mm) and shaken for 5 min using a shaker (HS 260 control NOL, IKA, Germany). Then the mold was compressed under a pressure of 250 bar for 30 min using a press machine (Automated X‐Press 3636, ATS, Canada). The resulted crystals were in the shape of round films. The crystal and mechanical‐annealed crystal of other amino acid were prepared using the same method.

### Fabrication of the Packaged Force Sensor

For the preparation of the PLA film, PLA was dissolved into dichloromethane (DCM) to a concentration of 50 mg mL^−1^. Then, PLA films with a thickness of 140 µm were obtained by casting the film in a glass dish and evaporating it at 25 °C for 12 h. PLA granules were dried at 60 °C in a vacuum oven overnight prior to being dissolved in DCM. For the preparation of the PLA–PAN electrode, one side of the PLA film was immersed in a mixed solution of sulfuric acid (1 m) and aniline (0.3 m). The whole system was bathed in ice water for 2 h. Then the PLA–PAN electrodes were taken out and washed with deionized water before being dried for 12 h.

For the fabrication of the force sensor, PLA glue was prepared by dissolving PLA into DCM to a concentration of 50 mg mL^−1^. Then, a square PLA film (10 mm × 10 mm) at a thickness of 0.3 mm with a hole (diameter: 8 mm) in the middle was prepared, and the crystal film (diameter: ≈8 mm; thickness: ≈0.35 mm) was filled into the hole to prepare the sandwiched layer. The PLA–PAN electrodes were cut into square films with dimensions of 10 mm × 10 mm. Then, the sandwiched layer was sandwiched between two PLA–PAN electrodes. All of the edges and surface of the device were then sealed using PLA glue and dried to finish the encapsulation of the device. For the sensor used in vivo, the dimension of the device was 8 mm × 8 mm, and the diameter of the crystal film was 6.5 mm. To improve the biodegradability, 0.1% w/v 8‐arm poly(ethylene glycol) amine hydrochloride salt (SUC) was mixed in to PLA solutions, and the crystal film was directly sandwiched between two PLA–PAN electrodes without the middle PLA film and sealed using PLA glue.

### Force and Motion Sensing Using Force Sensors

To measure the direct piezoelectric output of the sensors, various forces were applied to the device using a tensile machine (Instron 5944 with a 2 kN sensor). Polyimide enameled copper wires (diameter: 0.2 mm) were connected to the PAN layer of the device, and the output signal was recorded using a digital multimeter (Tektronix DMM6500 6½). For sensing in vitro, the force sensor was fixed on different surfaces (finger, wrist, and neck), and the output voltages were recorded.

For sensing in vivo, male Sprague–Dawley rats aged 8 weeks (250 ± 10 g) were used. The rats were anesthetized with isoflurane, and the hair of the chests or legs was removed. The force sensor (8 mm × 8 mm) was inserted through a 1.5‐cm skin incision at the interested locations. The injury was stitched with sutures (3‐0 Vicryl Plus, JNJ). Two polyimide enameled copper wires were exposed to air to connect with the digital multimeter. The output signals were recorded after implantation for 0, 1, 2, 3, 4, and 5 weeks.

### In Vitro and In Vivo Biocompatibility Evaluation

For the cell cytotoxicity determination, human breast cancer cells (MDA‐MB‐231) and adipose tissue‐derived stromal cells (ADMSCs) were used as the model cells. Generally, the cells were seeded in a 12‐well plate (5 × 10^4^ per well) in the presence of force sensors or mechanical‐annealed crystals. Cell incubation was performed at 37 °C and 5% CO_2_. After incubation for 24 h, a live/dead viability/cytotoxicity kit (calcein‐AM/propidium iodide double staining kit) was used to evaluate cell viability. After washing the wells twice with PBS solution, calcein AM and propidium iodide dye solution were mixed and added to each well. Then, the plate was incubated at 37 °C for 30 min before being washed with PBS (10 mm, pH = 7.4) three times. Finally, images were obtained using a laser confocal fluorescence microscopy (Olympus FV3000, Japan). For the cell morphology analysis, the cells were cultured as described above. After incubation for 24 h, the cytoskeleton and nucleus of the cells were stained with phalloidin and 4‐6‐diamidino‐2‐phenylindole (DAPI), respectively. The cell samples were fixed with 4% formaldehyde for 15 min and rinsed three times with precooled PBS. Then the cell samples were permeabilized with 0.25% v/v Triton X‐100 for 10 min at room temperature and rinsed three times with prewarmed PBS. Then non‐specific antibody binding was blocked by incubating the samples with 1% w/v bovine serum albumin (BSA) in PBST (0.1% v/v Triton X‐100 in PBS) at room temperature for 45 min. Next, the samples were incubated with phalloidin (phalloidin dilution 1:1000) in PBST with 1% BSA overnight at 4 °C, and rinsed twice with PBST and thrice with PBS. Then the samples were incubated with DAPI (1 µg mL^−1^, 1:500) in PBST with 1% BSA for 60 min at room temperature, and rinsed twice with PBST and thrice with PBS. At last, the samples were imaged using a laser confocal fluorescence microscopy (Olympus FV3000, Japan).

For the evaluation of acute inflammation, force sensors were implanted subcutaneously into male Sprague–Dawley rats through a 1.5‐cm skin incision at the center of the back. Then the incision was closed using interrupted sutures (3‐0 Vicryl Plus, JNJ). The force sensors were stored in a mixture of alcohol (75%) and ddH_2_O (25%) prior to implantation. After 1 day, the rats were sacrificed, and target subcutaneous regions were obtained and fixed in 10% formalin for 24 h before histological analysis (H&E staining) and immunofluorescent staining (TNF‐*α* and IL‐6). Blood samples were also collected for systemic toxicity tests based on hematological examination and blood biochemistry. For the long‐term systemic toxicity, force sensors were implanted subcutaneously for 6 weeks before the target subcutaneous regions, blood samples, and major organs were collected. Then, the hematological examination, blood biochemistry, histological analysis (H&E staining), and immunofluorescent staining were studied.

### In Vitro and In Vivo Biodegradation Evaluation

For a typical biodegradation test in vitro, the original dry weight of the force sensor was recorded as *W*_0_. Then the sensor (dimension: 10 mm × 10 mm, thickness: 0.7 mm) was immersed into 50 mL of SBF and incubated at 37 °C. After different times (1, 2, 3, 4, 5, and 6 weeks), the sensors were collected and the dry weights were recorded as *W_t_*. The remaining mass percentage at time *t* was determined as *ε* = *W_t_*/*W*_0_ × 100%.

For a typical biodegradation test in vivo, the force sensor (8 mm × 8 mm) was inserted between the skin and muscle through a 1.5‐cm skin incision at the center of the back of rats. After 1, 2, 4, and 6 weeks, the rats were sacrificed, and target subcutaneous regions were obtained and fixed in 10% formalin for 24 h before histological analysis (H&E staining). The sensors and surrounding tissues were embedded in resin to prevent the sensors from detaching during the H&E staining.

All animal studies were carried out in compliance with the regulations and guidelines of the Ethics Committee of Drum Tower Hospital affiliated to the Medical School of Nanjing University and conducted according to the Institutional Animal Care and Use Committee guidelines (Approval number: 2020AE01086).

### Statistical Analysis

IgorPro (Version 6.37) was used for statistical analysis. Data between two groups were analyzed by independent Student's *t*‐test. Significant values were presented as mean ± standard deviation. NS: *p* > 0.05 was considered not significant and *: *p* < 0.05 was considered significant.

## Conflict of Interest

The authors declare no conflict of interest.

## Supporting information

Supporting Information

Supporting Information

Supplemental Movie 1

Supplemental Movie 2

## Data Availability

The data that support the findings of this study are available from the corresponding author upon reasonable request.
