# Supplementary material for: Boosting the Piezoelectric Sensitivity of Amino Acid Crystals by Mechanical Annealing for the Engineering of Fully Degradable Force Sensors
Source: Adv Sci (Weinh). 2023 Feb 12;10(11):2207269. doi: 10.1002/advs.202207269 (PMC10104669; doi:10.1002/advs.202207269)
Supplement: Supplementary file 1 — Supporting Information [file ADVS-10-2207269-s003.pdf]

Supporting Information

Boosting the piezoelectric sensitivity of amino acid crystals by mechanical annealing for the engineering of fully degradable force sensors

Yuanqi Cheng, Juan Xu, Lan Li, Pingqiang Cai, Ying Li, Qing Jiang, Wei Wang*, Yi Cao*, and Bin Xue**

Experimental Section

Materials

L-Isoleucine, L-histidine and Triton X-100 were purchased from Sigma-Aldrich, Shanghai, China. Polylactic acid (PLA, Mw: 80 kDa) was purchased from Macklin, Shanghai, China. 8-arm Poly(ethylene glycol) amine hydrochloride salt(SUC) was purchased from SINOPEG, Xiamen, China. Eight-week-old male Sprague-Dawley rats were obtained from BiKai Company, Nanjing, China. The calcein-AM and propidium iodide (PI) double staining kit (cat: KGAF001, Keygen, China), phalloidin (Alexa Fluor™ 647) and 4-6-diamidino-2-phenylindole (DAPI, D-1306) were purchased from Invitrogen™, USA. The cell culture medium AMEM and DMEM (cat: 310-010-CL and cat: 319-051-CL) were purchased from Wisent, Nanjing, China. The culture supplement foetal bovine serum (FBS) (cat: 10091148) was purchased from Gibco, USA. Human breast cancer cells (MDA-MB-231) and human adipose derived stem cells (ADMSCs) were kindly provided by Stem Cell Bank, Chinese Academy of Sciences. All other chemical reagents, unless otherwise stated, were purchased from Shanghai Aladdin Biochemical Technology Co., Ltd. (China). All reagents were used without further purification.

Atomic force microscopy (AFM) based nanoindentation

Atomic force microscopy (AFM) based nanoindentation experiments were performed using a commercial AFM (JPK, Nanowizard IV, Berlin, Germany). The crystals were spread on mica substrates and then blown with nitrogen to remove the loose samples. Typically, the cantilever was moved to the surface of the crystal, and an area with the size of 10 μm × 10 μm was scanned in tapping mode to find the flat area. Then, nanoindentation was performed on the flat area (scan area: 5 μm × 5 μm) in QI mode (conditions: pixels: 256 × 256; Z length: 0.05

μm; extend and retract speed: 30 μm s^{-1}; Z resolution: 80000 Hz; maximum loading force: 800 nN). RTESPA-525 cantilevers (Bruker Company, half-open angle of the pyramidal face of θ: < 10°, tip radius: ~10 nm, spring constant: ~200 N m^{-1}) were used in all experiments. The cantilever was extended to the surface of the crystal and retracted. The force-displacement curves during the process were recorded. The Young's modulus of the crystals could be calculated by fitting the extending curve with the Hertz model (1).

$$F = \frac{4}{3}\frac{E}{(1-\nu^2)}\sqrt{R}\delta^{3/2} \quad (1)$$

Here F corresponds to the force, δ corresponds to the depth of the crystal pressed by the cantilever tip, R is the radius of the tip, E is the Young's modulus of the crystals and ν is the Poisson ratio (ν = 0.3). The point stiffness was determined as the normal force divided by the deformation of the sample and calculated from the force-displacement curves after deducting the deformation of the cantilever. More than six regions and 1-2 flat areas at each location were randomly selected to perform the nanoindentation. At least three cantilevers were used in the experiments to exclude a tip dependency of the results. All the data were analysed and the two-dimensional diagrams were reconstructed using JPK data processing 7.0.46 software (JPK company).

Thermogravimetric analysis (TGA) and differential scanning calorimetry (DSC)

The TGA and DSC experiments were performed using a synchronous thermal analyzer (METTLER TOLEDO TGA/DSC-1, Switzerland) at a scanning rate of 5 °C min^{-1} in a flow of dry nitrogen over a range of 30 to 600 °C.

Powder X-ray diffraction (XRD)

XRD spectra of all the crystals were recorded using an X-ray powder diffractometer (D8 Advance, BRUKER AXS GmbH) at 25 °C. The scan speed was 0.5 s^{-1} and the scan range was 5°- 80°.

Single crystal X-ray diffraction

The single crystal of isoleucine was coated in Paratone Oil (Hampton Research) and mounted on MiTeGen cryo-loops before being flash frozen in liquid nitrogen gaseous stream (Oxford Cryostream). Diffraction data for the crystal were collected on a Rigaku XtaLabPro diffractometer with Cu radiation (λ= 1.54184 Å). Diffraction data were collected on a Rigaku

XtaLabPro diffractometer with Mo radiation (λ= 0.71073 Å). Data were integrated and reduced using the 'CrysAlisPro (Rigaku OD, 2019)'.

Processing and structural refinement of crystal data

The diffraction data were processed using CrysAlisPro (Rigaku OD, 2019)'. The structure was solved by direct methods using SHELXT-2014/5.[1] The refinements were performed with SHELXL-2016/4 and weighted full-matrix least-squares against $|F^2|$ using all data. Atoms were refined independently and anisotropically, with the exception of hydrogen atoms, which were placed in calculated positions and refined in a riding mode. Crystal data collection and refinement parameters are shown in Table S1 and the complete data can be found in the cif file as supplementary information.

Scanning electron microscopy (SEM)

SEM images were obtained using a Quanta scanning electron microscope (Quanta 200, FEI) at 15 kV. The samples were dried and coated with Pt prior to the measurement.

Piezoelectric coefficient measurement

The piezoelectric constants in the normal direction of the films ($d_{\perp}$) were measured using a quasistatic d_{33} piezometer (ZJ-3, IACAS, China) under a dynamic force of 0.36 N.

Mechanical property of PLA-PAN films

The tensile stress-strain measurements of the PLA-PAN films were performed using a tensile-compressive tester (Instron-5944 with a 2 kN sensor) in air at room temperature. The strain rate of stretching was maintained at ~10 mm min^{-1}. The Young's modulus corresponded to the approximate linear fitting value under 10% strain deformation.

Figures

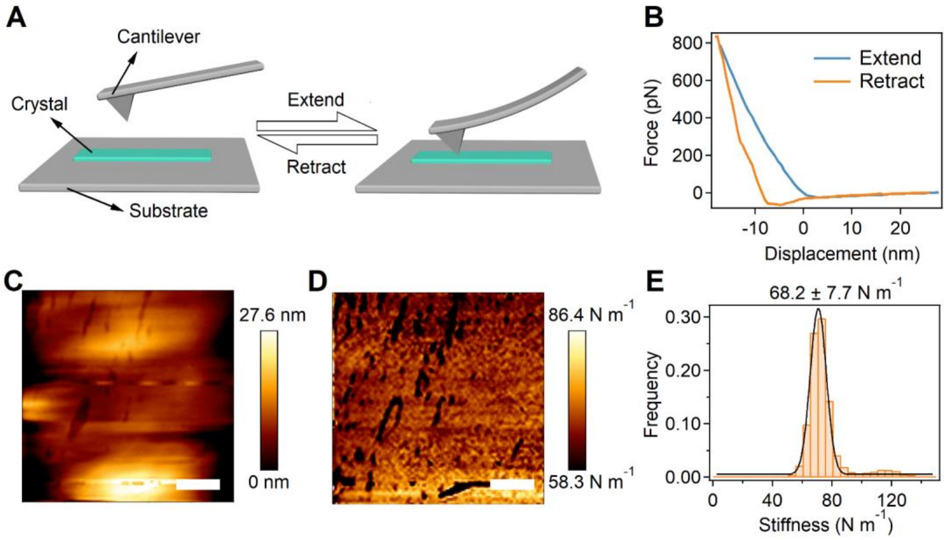

Figure S1. AFM-based nanoindentation of single isoleucine crystals. (A) Schematic of AFM-based nanoindentation on single isoleucine crystals. (B) Typical force-displacement curves from the AFM-based nanoindentation experiments. The modulus was determined by fitting the extend-displacement curve with the Hertz model. (C and D) Topographic height (C) and stiffness (D) maps of the isoleucine crystal. Scale bar =1 μm. (E) Statistical point-stiffness distribution of the isoleucine crystal.

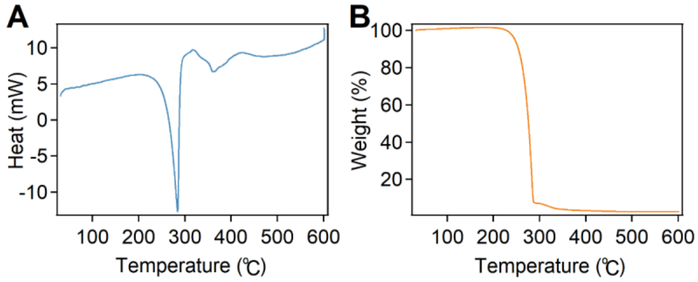

Figure S2. DSC (A) and TG (B) spectra of isoleucine crystals. The decomposition temperature reached 283 °C.

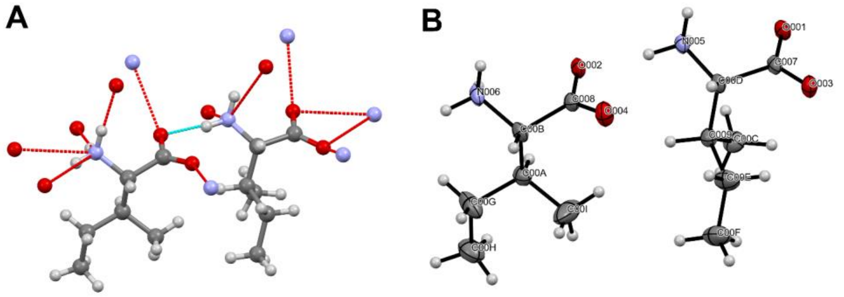

Figure S3. Asymmetric unit and Oak Ridge thermal ellipsoid plot (ORTEP) diagram of the isoleucine crystal. (A) Asymmetric unit of the isoleucine crystal showing H-bonding between neighbouring isoleucine molecules. (B) ORTEP diagram of isoleucine with the thermal ellipsoids set at 50% probability.

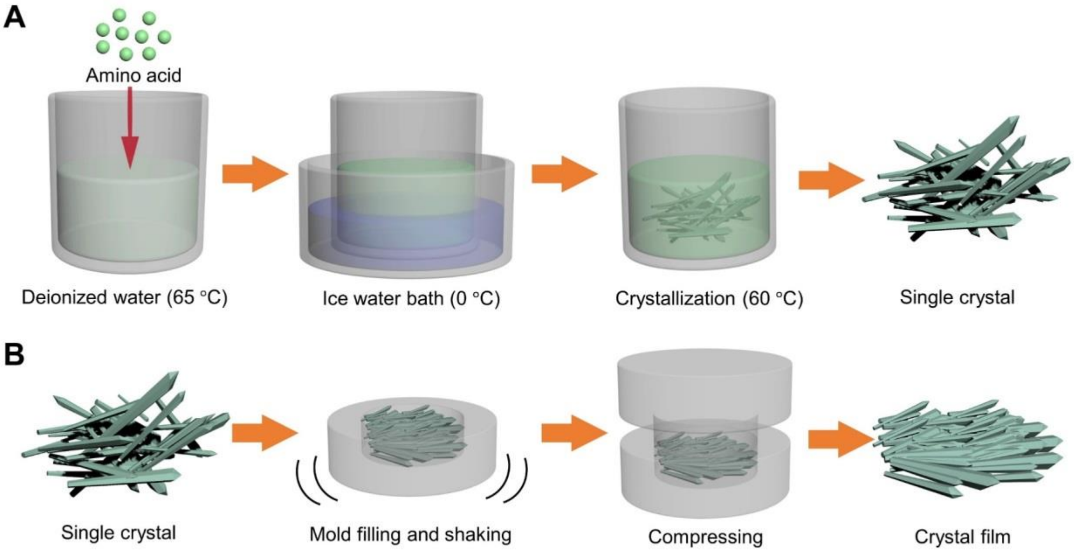

Figure S4. Schematic diagram for the preparation of amino acid crystal and crystal films. (A) Schematic diagram for the preparation of amino acid crystal. (B) Schematic diagram for the preparation of crystal film.

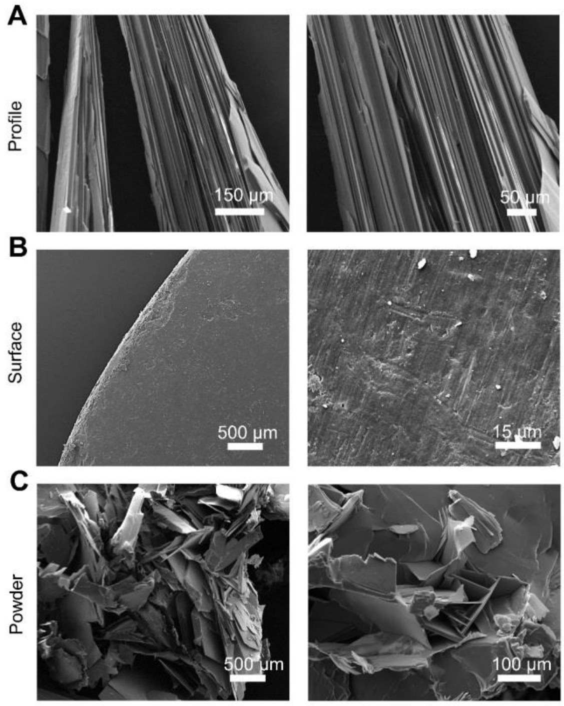

Figure S5. SEM images of the mechanical-annealed isoleucine crystal and the powder formed isoleucine crystal. (A) SEM images of the profile of the isoleucine crystal with mechanical annealing. (B) SEM images of the surface of the isoleucine crystal with mechanical annealing. (C) SEM images of the powder formed isoleucine crystal without mechanical annealing.

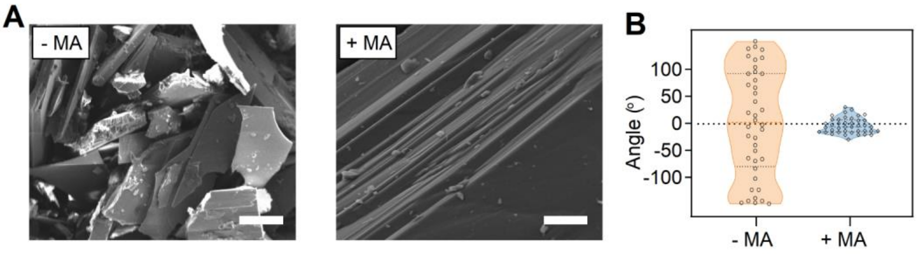

Figure S6. Mechanical annealing of histidine crystals. (A) SEM images of histidine crystals without (- MA) and with (+ MA) mechanical annealing. Scale bar = 25 µm. (B) Distribution of angles between the crystal's long axis and the horizontal XY plane (the plane perpendicular to the applied force) without (- MA) and with (+ MA) mechanical annealing.

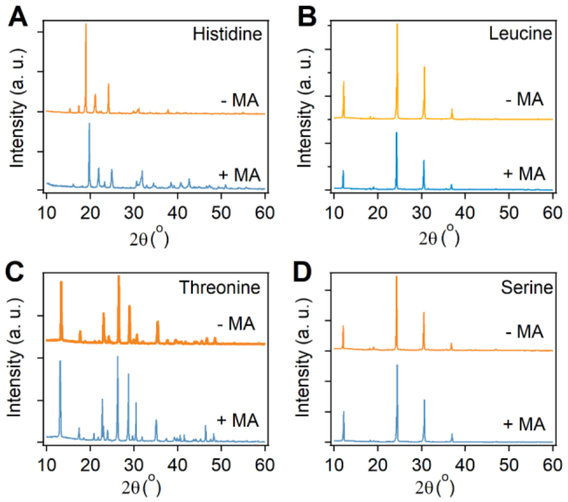

Figure S7. XRD of histidine (A), leucine (B), threonine (C) and serine (D) crystals without (-MA) and with (+ MA) mechanical annealing.

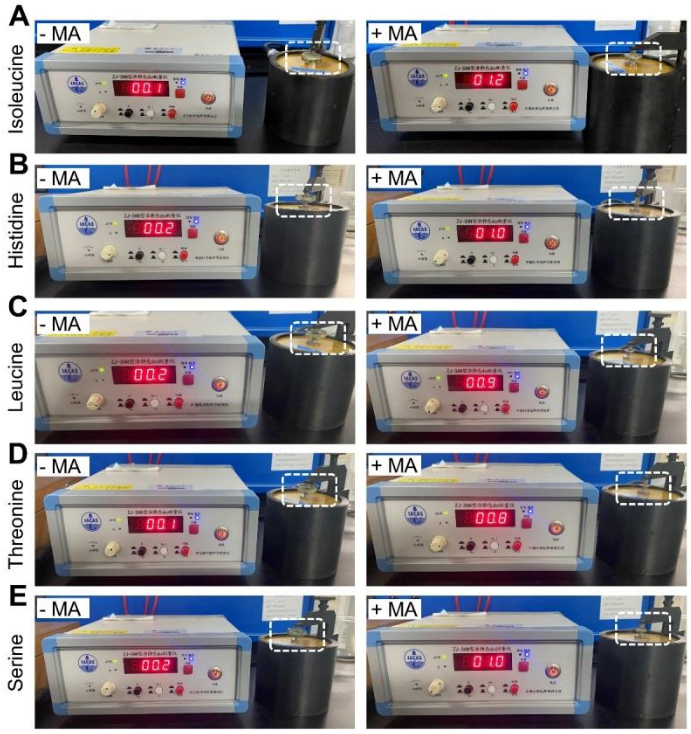

Figure S8. Quasistatic piezoelectric constants ($d_{\perp}$) of the isoleucine (A), histidine (B), leucine (C), threonine (D) and serine (E) crystals with (left) and without (right) mechanical annealing. The samples are highlighted with dashed boxes.

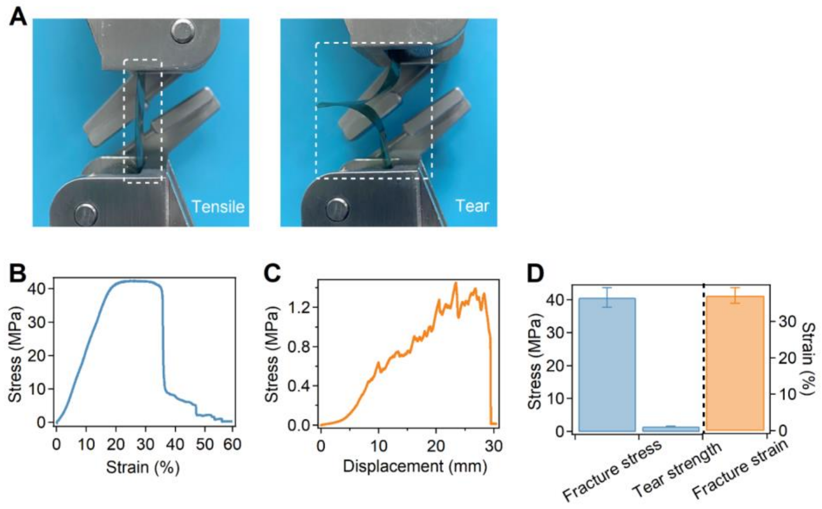

Figure S9. Mechanical properties of the PLA-PAN layers. (A) Photographs of typical tensile and tear experiments of the PLA-PAN layer. (B) Typical stress-strain curve for tensile measurements of the PLA-PAN layer. (C) Typical stress-displacement curve for tear measurements of the PLA-PAN layer. (D) Summarized facture stress and fracture strain for tensile measurements, and tear strength for tear measurements.

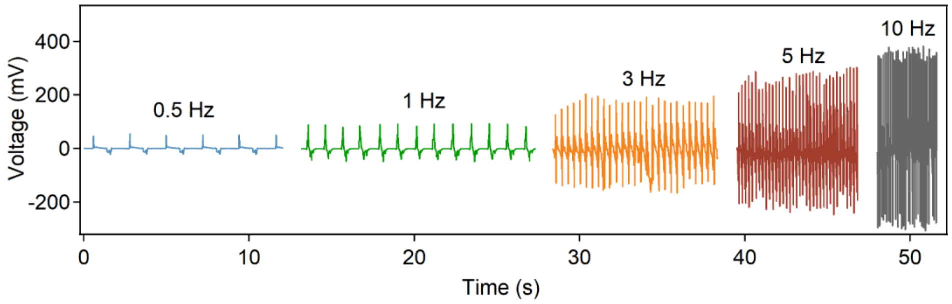

Figure S10. Open-circuit voltages of packaged force sensors prepared with the mechanical-annealed isoleucine crystal under applied forces (10 N) at different frequencies (0.5-10 Hz).

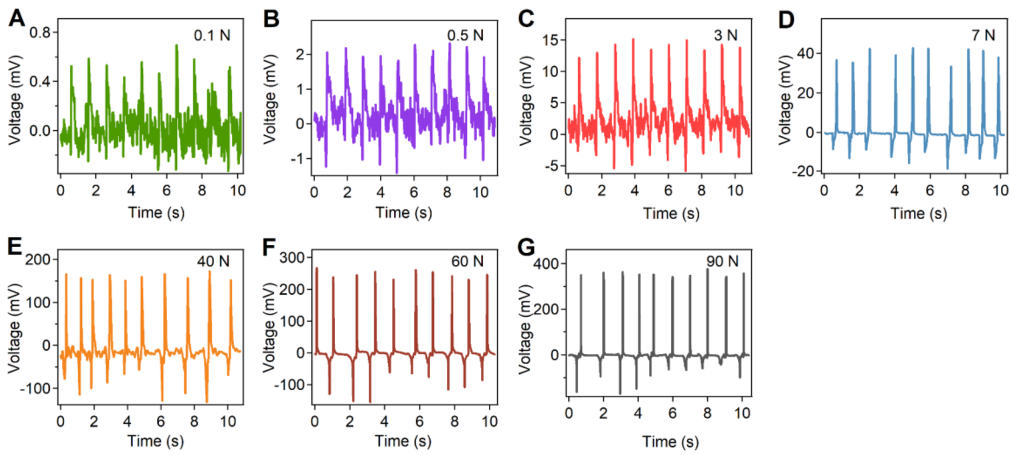

Figure S11. Open-circuit voltage of force sensors containing the mechanical-annealed isoleucine crystal film under applied forces of 0.1 (A), 0.5 (B), 3 (C), 7 (D), 40 (E), 60 (F), and 90 (G) N.

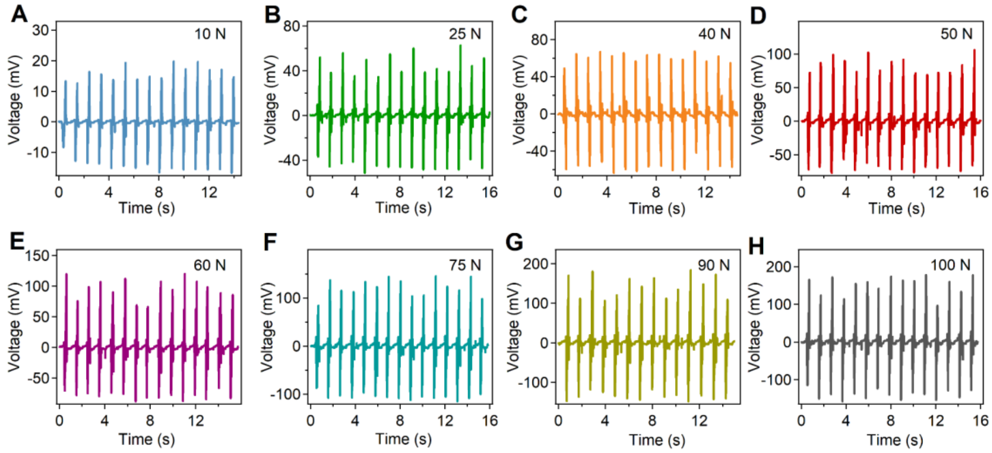

Figure S12. Open-circuit voltages of the packaged force sensors prepared using the unaligned isoleucine crystal powders under forces of 10 (A), 25 (B), 40 (C), 50 (D), 60 (E), 75 (F), 90 (G), and 100 (H) N.

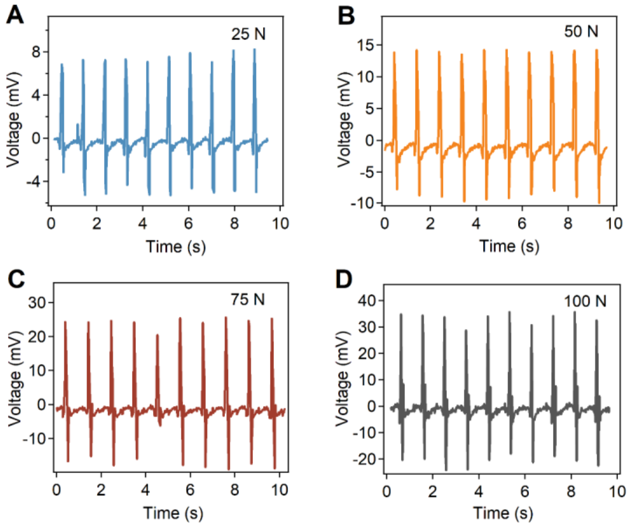

Figure S13. Open-circuit voltages of the force sensor prepared without crystal under forces of 25 (A), 50 (B), 75 (C), and 100 (D) N.

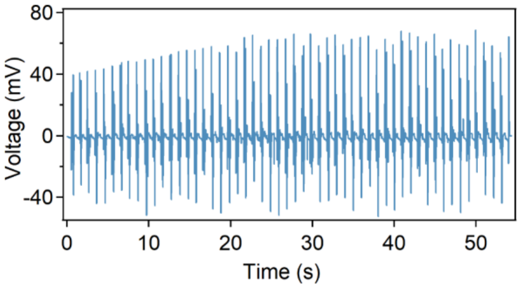

Figure S14. Open-circuit voltages of the force sensor prepared using the unaligned isoleucine crystal powders under continuous impulse impact (force: 40 N). The sensing stability was affected by the permanent deformation of the powder formed crystal inside the sensor.

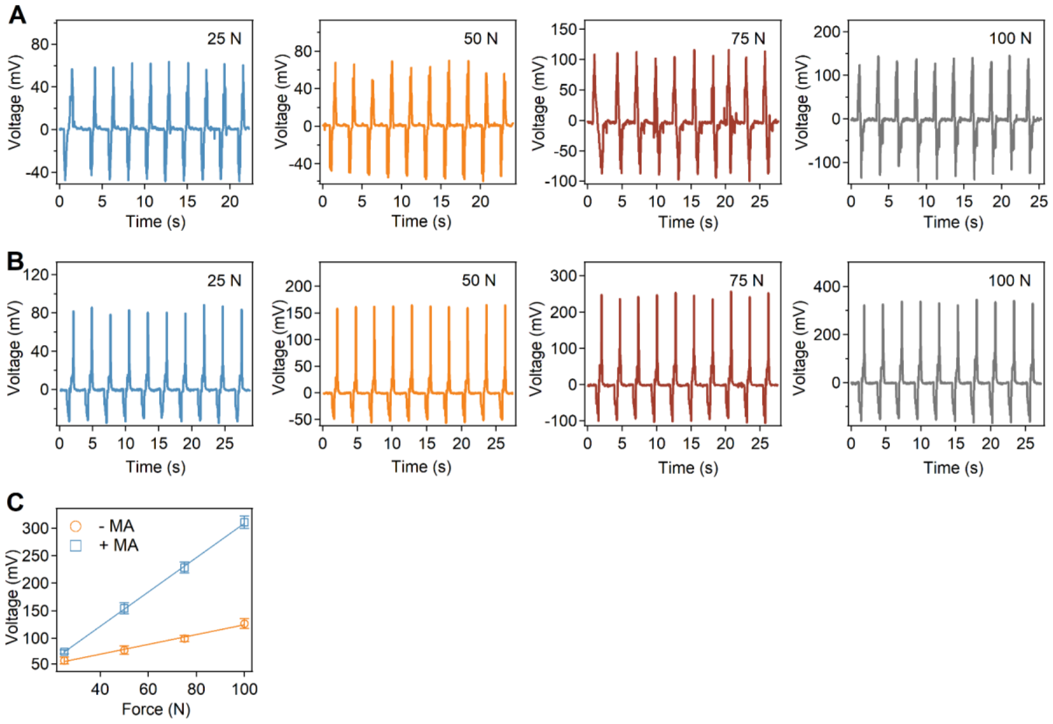

Figure S15. Force sensing of the sensor prepared using the histidine crystal with and without mechanical annealing. (A) Open-circuit voltage of the sensor prepared using unaligned histidine crystal powders under different forces (25, 50, 75 and 100 N). (B) Open-circuit voltage of the sensor prepared using the mechanical-annealed histidine crystal film under different forces (25, 50, 75 and 100 N). (C) Linear fitting of the open-circuit voltage of the force sensors as a function of the applied force. The sensors were prepared using the mechanical-annealed histidine crystal film (+ MA) and unaligned histidine crystal powders (-MA). Values represent the mean and standard deviation (n = 3 independent samples).

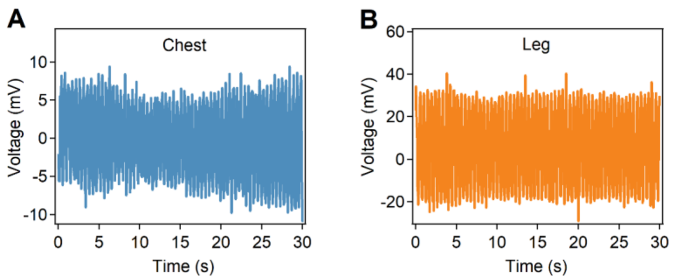

Figure S16. Bioelectrical signals (voltage vs time) from the chest (A) and leg (B) of rats. The distinct signals from those recorded by force sensors indicated that the open-circuit voltages of the sensors were indeed caused by respiration and leg stretching.

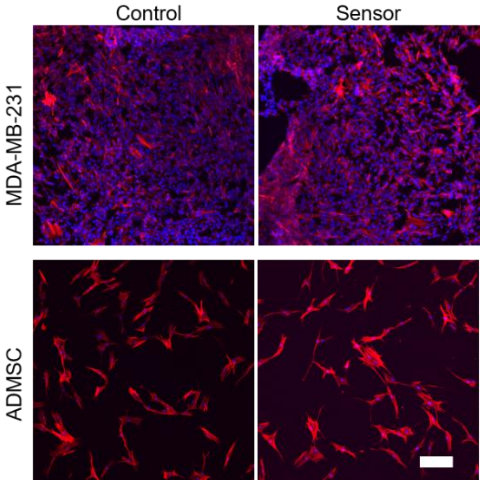

Figure S17. Cellular morphology and nucleus of MDA-MB-231and ADMSC cells stained with phalloidin (red) and DAPI (blue) after being cultured for 48 h in the absence (Control) and presence (Sensor) of the packaged force sensors. Scale bar = 100 μm.

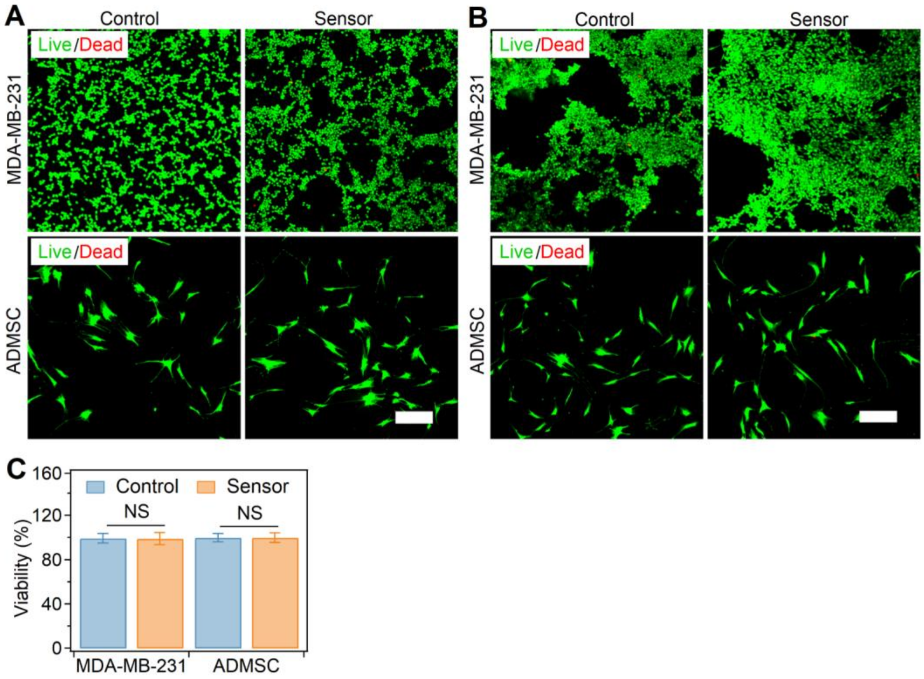

Figure S18. Viabilities determination of MDA-MB-231 and ADMSC cells after being cultured for 24 and 48 h. (A-B) Live/dead staining of MDA-MB-231 and ADMSC cells after being cultured for 24 h (A) and 48 h (B) in the absence (Control) and presence (Sensor) of the packaged force sensors. The living (green) and dead (red) cells were stained with the Calcein-AM/PI Double Staining Kit. Scale bar = 100 μm. (C) Cell viabilities of MDA-MB-231 and ADMSC cells cultured in the absence and presence of the packaged force sensors after 48 h. Statistical significance was determined by a two-tailed t-test. NS, not significant.

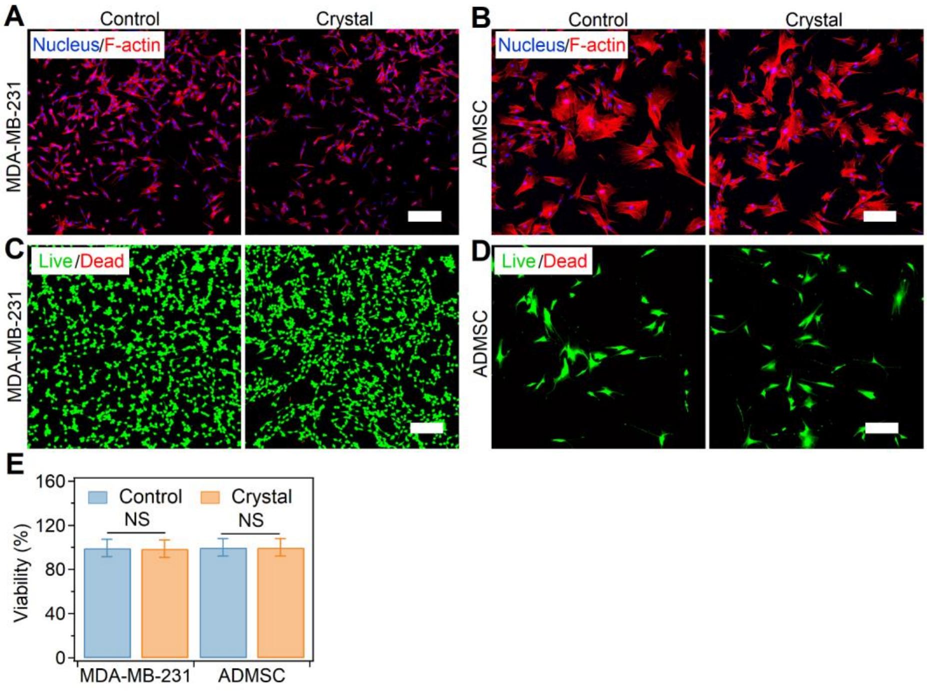

Figure S19. Cytotoxicity of the mechanical-annealed isoleucine crystals. (A and B) Cellular morphology and nucleus of MDA-MB-231 (A) and ADMSC (B) cells stained with phalloidin (red) and DAPI (blue) after being cultured for 24 h in the absence (Control) and presence (Crystal) of the mechanical-annealed isoleucine crystals. (C and D) Live/dead staining of MDA-MB-231 (C) and ADMSC (D) cells after being cultured for 24 h in the absence (Control) and presence (Crystal) of the mechanical-annealed isoleucine crystals. The living (green) and dead (red) cells were stained with the Calcein-AM/PI Double Staining Kit. Scale bar = 100 μm. (E) Cell viability of MDA-MB-231 and ADMSC cells cultured in the presence of the mechanical-annealed isoleucine crystal after 24 h. Statistical significance was determined by a two-tailed t-test. NS, not significant.

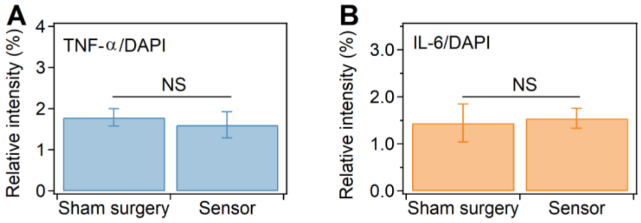

Figure S20. Relative intensity of TNF-α/DAPI (A) or IL-6/DAPI (B) in the images for the sham surgery and sensor implanted groups. Values represent the mean and the standard

deviation (n = 5). Statistical significance was determined by a two-tailed t-test. NS, not significant.

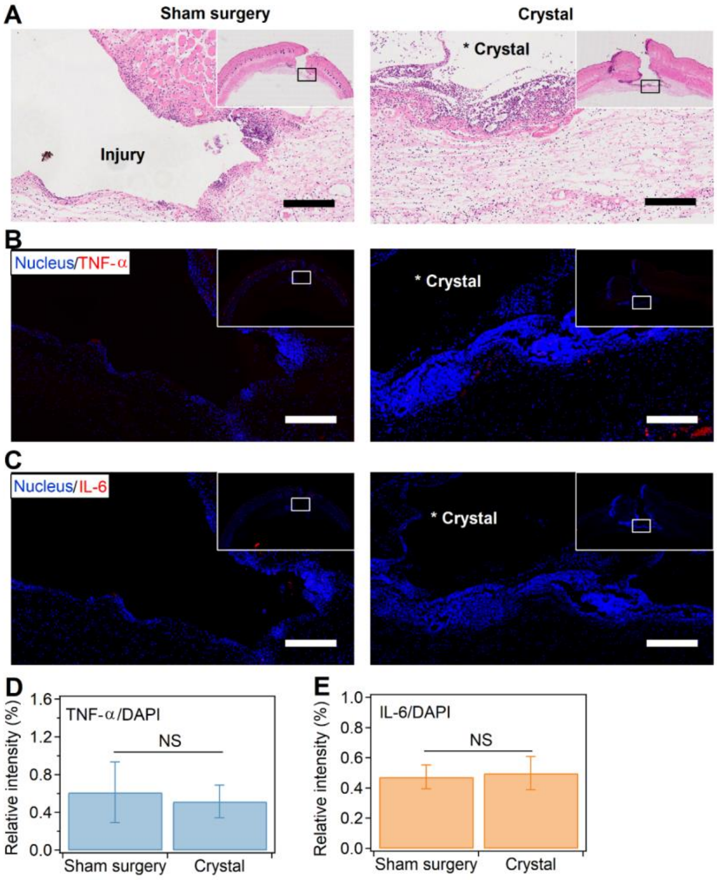

Figure S21. In vivo biocompatibility of mechanical-annealed isoleucine crystals based on dorsal subcutaneous implantations in a rat model. (A) Representative histological images stained with hematoxylin and eosin (H&E) in vivo after subcutaneous implantation for 1 day. * represents the location of the mechanical-annealed isoleucine crystal. Scale bar = 250 µm. (B) Representative immunostaining images identified by TNF-α (red). Cell nuclei are indicated by DAPI (blue). Scale bar = 250 µm. (C) Representative immunostaining images identified by IL-6 (red). Cell nuclei are indicated by DAPI (blue). Scale bar = 250 µm. (D and E) Relative intensity of TNF-α/DAPI (D) or IL-6/DAPI (E) in the images for sham surgery and crystal implanted groups. Values represent the mean and the standard deviation (n = 5). Statistical significance was determined by a two-tailed t-test. NS, not significant.

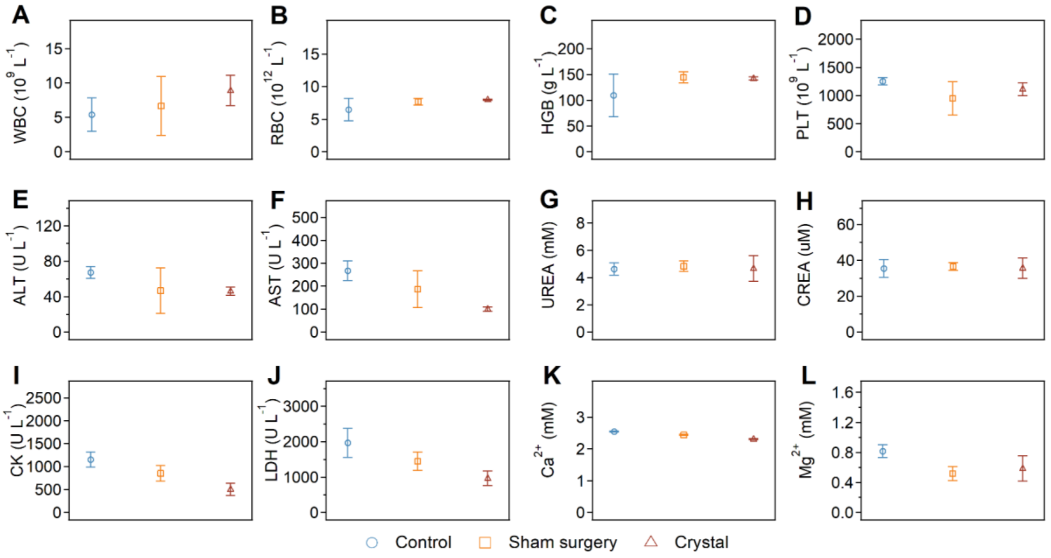

Figure S22. Hematological examination (A to D) and blood biochemistry (E to L) of the rats after subcutaneous implantation of the mechanical-annealed isoleucine crystals for 1 day. The blood of rats without any surgery was set as the control. WBC: white blood cell count, RBC: red blood cell count, HGB: hemoglobin, PLT: platelets, ALT: alanine transferase, AST: aspartate transferase, UREA: urea, CREA: creatinine, CK: creatine kinase, LDH: lactate dehydrogenase. Values represent the mean and the standard deviation (n = 3 independent animals).

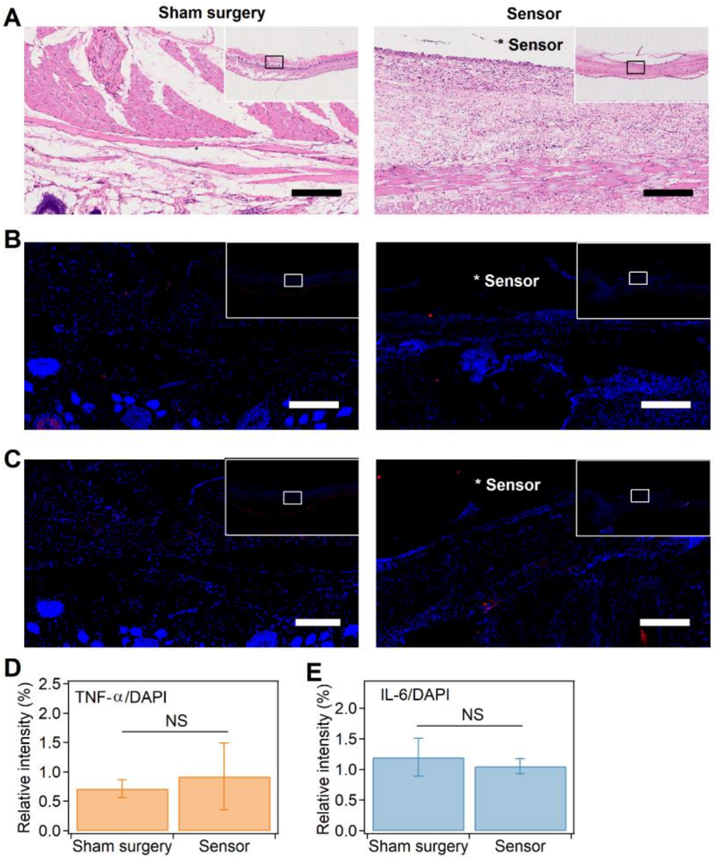

Figure S23. Histological and immunofluorescence analysis of the dorsal subcutaneous implantation of force sensors in a rat model after 6 weeks. (A) Representative histological images stained with hematoxylin and eosin (H&E) in vivo after subcutaneous implantation for 6 weeks. * represents the location of the force sensor. Scale bar = 250 μm. (B) Representative immunostaining images identified by TNF-α (red). Cell nuclei are indicated by DAPI (blue). Scale bar = 250 μm. (C) Representative immunostaining images identified by IL-6 (red). Cell nuclei are indicated by DAPI (blue). Scale bar = 250 μm. (D and E) Relative intensity of TNF-α/DAPI (D) or IL-6/DAPI (E) in the images for the sham surgery and sensor implanted groups. Values represent the mean and the standard deviation (n = 5). Statistical significance was determined by a two-tailed t-test. NS, not significant.

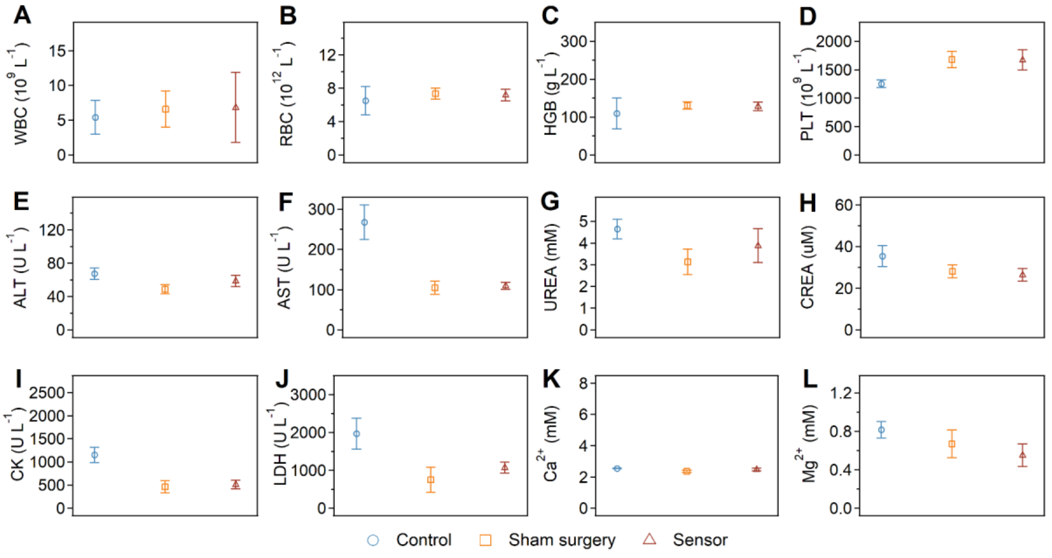

Figure S24. Hematological examination (A to D) and blood biochemistry (E to L) of the rats after subcutaneous implantation of force sensors for 6 weeks. The blood of rats without any surgery was set as the control. WBC: white blood cell count, RBC: red blood cell count, HGB: hemoglobin, PLT: platelets, ALT: alanine transferase, AST: aspartate transferase, UREA: urea, CREA: creatinine, CK: creatine kinase, LDH: lactate dehydrogenase. Values represent the mean and the standard deviation (n =3 independent animals).

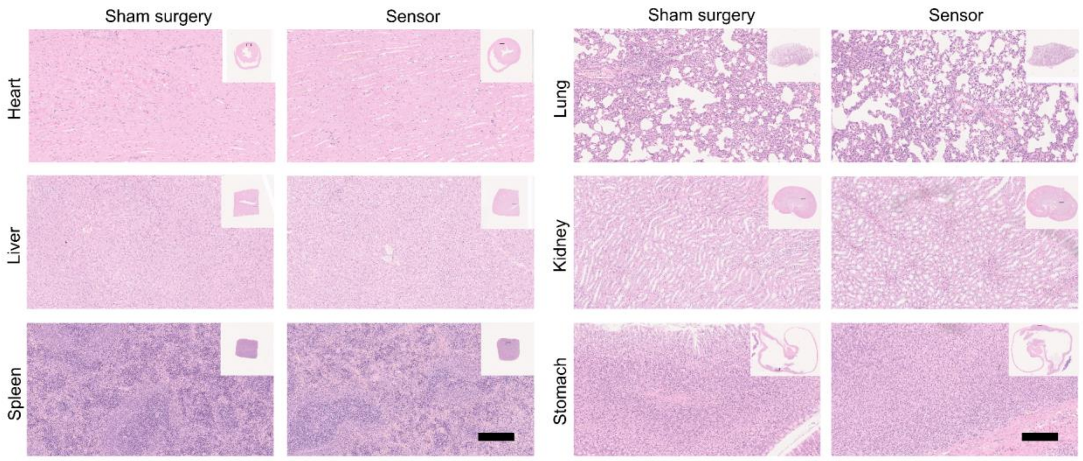

Figure S25. Representative H&E staining of major organs (the heart, liver, spleen, lung, kidney and stomach) after subcutaneous implantation of the force sensor for 6 weeks in the backs of Sprague Dawley rats. The organs from the rats that underwent sham surgery were used for comparison.

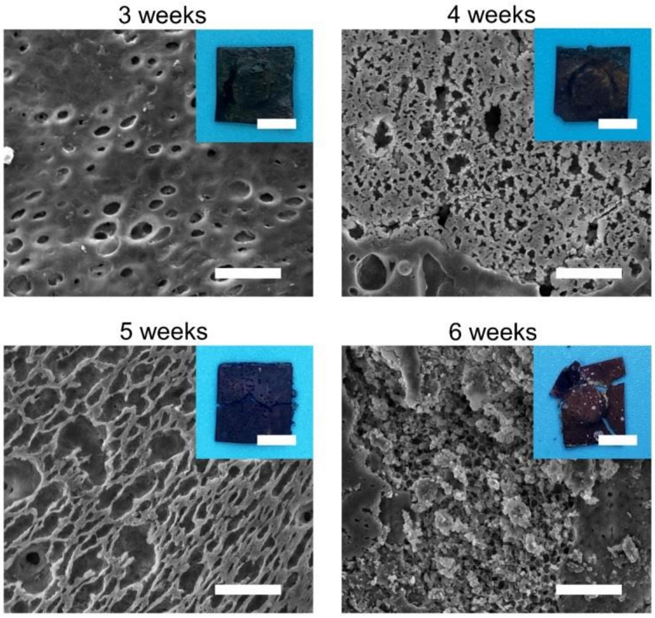

Figure S26. SEM images of the packaged force sensors after incubation in SBF for different times (3, 4, 5 and 6 weeks). Insets are the corresponding photographs of the force sensors. Scale bars of the SEM images and insets are 50 μm and 5 mm, respectively.

Table S1. Data collection and refinement statistics for X-ray crystallography.

**Crystal data**	**Isoleucine**
**Chemical formula**	$C_6H_{13}NO_2$
Mr	131.17
**Crystal system**	Monoclinic
**Space group**	$P2_1$
a **(Å)**	9.6818(2)
b **(Å)**	5.28470(10)
c **(Å)**	13.9630(3)
**α (°)**	90
**β (°)**	96.031(2)
**γ (°)**	90
V **(Å$_3$)**	710.468
Z	4
**Mυ (mm^{-1})**	0.748
**Temperature (K)**	170
**Diffractometer**	Rigaku XtaLab^{Pro}

**Wavelength λ= (Å)**	1.54184
**Absorption correction**	multi-scan
T_{min}, T_{max}	0.254,1.000
No. reflections, (unique)	3078 (1710)
**θ_{max} (°)**	78.932
R_1, wR_2 **(all data)**	0.0771,0.1933
**No. of reflections**	5602
**No. of parameters**	170
**H-atom treatment**	H-atom parameters mixed

Movie S1.

In vivo sensing of rat respiration.

Movie S2.

In vivo sensing of gentle leg stretching.

Reference

[1] G. Sheldrick, Structure Determination (SHELXS) and ii. Refinement (SHELXL-2013); University of Gottingen: Germany, **2013**.
